# The Childbearing sense of coherence scale (CSOC-scale): development and validation

**DOI:** 10.1186/s12889-024-19109-1

**Published:** 2024-06-17

**Authors:** Bingbing Li, Meizhen Zhao, Zining Zhu, Huimin Zhao, Xi Zhang, Jingxin Wang, Tieying Zeng, Mengmei Yuan

**Affiliations:** 1 Department of Nursing, Tongji Hospital, Tongji Medical College, Huazhong University of Science and Technology, 1095 Jiefang Avenue, Wuhan, 430030 China; 2 School of Nursing, Tongji Medical College, Huazhong University of Science and Technology, 13 Hangkong Road, Wuhan, 430030 China; 3 College of Nursing, Shanxi University of Chinese Medicine, Jinzhong, 030619 China

**Keywords:** Salutogenesis, Instrument development, Psychometrics, The childbearing sense of coherence scale, Pregnancy, Couples

## Abstract

**Background:**

the salutogenic theory is essential to explain an individual’s ability to maintain health during the perinatal period. While previous studies mainly focused on the perspectives from a family-level orientation and a global orientation, the purpose of the present study was to develop and validate a scale, the childbearing sense of coherence scale (CSOC-scale) from the individual’s perceptions of the stresses, resources, and meaningfulness of childbearing.

**Methods:**

A total of 3 separate studies contributed to the development of the CSOC-scale between July 2022 and February 2023. In study 1, the initial item pool based on the conceptual framework of the childbearing sense of coherence and the salutogenic theory was developed. Delphi expert consultation was conducted to revise and improve items. Studies 2 and 3 were cross-sectional studies. In study 2, item analysis and explore factor analysis (EFA) (*N* = 351 for women, *N* = 256 for men) were used to screen items. In study 3, confirmatory factor analysis (CFA) and reliability analysis (*N* = 366 for women, *N* = 308 for men) were used to test the fit indices and reliability of the final scale.

**Results:**

final analysis suggested the CSOC-scale includes three factors, consisting of 13 items. Confirmatory factor analysis demonstrated good model fit (*χ*^*2*^ = 157.448, *df* = 62, *χ*^*2*^*/ df* = 2.539, RMSEA = 0.065, CFI = 0.974, TLI = 0.968, SRMR = 0.029 for women; *χ*^*2*^ = 181.363, *df* = 62, *χ*^*2*^*/ df* = 2.925, RMSEA = 0.079, CFI = 0.968, TLI = 0.960, SRMR = 0.033 for men) and high factor loadings (from 0.751 to 0.929 for women; from 0.746 to 0.947 for men). Internal consistency (Cronbach’s α ranging from 0.895 to 0.933 for women and 0.881 to 0.945 for men in three dimensions; Cronbach’s α was 0.919 for women and 0.821 for men in the entire instrument) and split-half reliability (Spearman-Brown coefficients ranging from 0.876 to 0.921 for women and 0.841 to 0.937 for men in three dimensions; Spearman-Brown coefficient was 0.744 for women and 0.785 for men in the entire instrument) were excellent.

**Conclusions:**

the CSOC-scale has robust psychometric properties. It is reliable and valid in evaluating the childbearing sense of coherence in women and men during pregnancy. Utilisation of this scale can help healthcare professionals understand the health maintenance competencies of couples during the transition of parenthood and provide health promotion services from a salutogenic perspective.

**Supplementary Information:**

The online version contains supplementary material available at 10.1186/s12889-024-19109-1.

## Introduction

Pregnancy and childbirth are natural processes, but they are the singular period for women and their husbands in the life cycle. Women and men will experience intense transformation during the perinatal period, which occurs in physiological status and psychosocial role [1–3]. Women must adjust to physiological changes such as the body’s structure, image, function, and hormones [4, 5]. Both women and men might encounter changed roles in the workplace, social activities, and family, expanded financial strain, and a surge in resource needs [6, 7]. If they cannot adapt to these changes in the transition to parenthood, then their health will be affected to a great extent. Previous studies have revealed that for experienced or first-time parents, the transition to parenthood demands an adaptation and creates stress for both women and men [8]. Couples during the perinatal period are vulnerable to suffering from health problems such as impaired sleep quality, anxiety, and depression [2, 9–11].

Recently, researchers have demonstrated the importance of salutogenesis in maintaining parental health during the perinatal period [12–15]. According to salutogenesis, the individual’s health moves on a continuum from entirely healthy to completely unhealthy. Stress can promote health when stressors are actively managed; when one’s resources and the ability to use them are insufficient to meet demand, the tension becomes stress that moves one toward the unhealthy end of the continuum and vice versa [16]. The ability of people to mobilise resources to promote health could be evaluated by the sense of coherence (SOC). The SOC explains the extent to which people perceive their lives and stressors as comprehensible, manageable, and meaningful [16]. Both expectant parents and parents are also in great demand of resources due to the changes and plights incurred, and singularly exclusive during the transition to parenthood period. Their ability to mobilise resources matters a lot, as it is crucial to smooth out the problems ahead of them. The childbearing sense of coherence (CSOC) is a concept developed from the salutogenic theory [17]. The CSOC is defined as the individual’s perceptions of childbearing, reflecting the individual’s perceptions of childbearing stresses, resources, and meaningfulness [17]. The childbearing sense of coherence includes three dimensions: comprehensibility of childbearing, manageability of childbearing, and meaningfulness of childbearing; comprehensibility of childbearing refers to the extent to which the individual regards the stimuli from the internal and external environments during childbearing as reasonable and acceptable; manageability of childbearing refers to the extent to which the individual utilises internal and external resources to cope with challenges from childbearing; meaningfulness of childbearing refers to the extent to which the individual recognises the meaningfulness of everything experienced during childbearing and is willing to put effort into it [17]. According to the concept of the childbearing sense of coherence, compared with the lower levels of counterparts, the individual who has higher levels of childbearing sense of coherence would be able to understand and accept the changes that come with childbearing better, utilise more sufficient resources to cope with challenges encountered during the parenthood transition and perceive more meaningfulness of childbearing [17]. The childbearing sense of coherence could assess an individual’s ability to maintain their health during the perinatal period [17]. Therefore, understanding the level of parental childbearing sense of coherence is essential for developing intervention strategies for health promotion among couples in the perinatal period.

Current measures developed from the salutogenic theory including the Family Sense of Coherence Scale (FSOC) and Sense of Coherence Scale (SOC), which were developed by Antonovsky and colleagues to measure the sense of coherence in levels of family life and individual life, respectively [18, 19]. The items of the two scales are constructed to assess a family level and individual level of global orientation in three dimensions (comprehensible, manageable, and meaningful). The FSOC scale consists of family situation descriptions of what a family might encounter and one’s feelings. The SOC scale consists of general situation descriptions of what individual might encounter and their feelings. Since childbearing is context-specific and a particular part of family life and personal life, the childbearing context-based scale is more appropriate for individuals during the perinatal period. According to the salutogenic theory, the childbearing sense of coherence has the potential to influence the individual’s perception and adjustment to stressors and promote positive adaptation during transition to parenthood. However, there is no measure to evaluate the childbearing sense of coherence. Despite the importance of the salutogenic lens, existing measurements have ignored the childbearing-related aspect of it. Therefore, this study aimed to develop and validate the childbearing sense of coherence scale (the CSOC-scale).

## Methods

This research consisted of three separate studies. Study 1 aimed to develop the initial CSOC scale. Study 2 aimed to test the psychometric properties of the scale. Study 3 aimed to evaluate the validity and reliability of the final scale. The study protocol was approved by the Ethics Committee of Tongji Hospital (reference number: IRB20220705). Informed consent from all participants was obtained, after the explanation of the purpose and procedure of the study as well as the potential risks and rights for them.

### Study 1: Initial scale development

#### Item selection

The concept model of the childbearing sense of coherence was created based on the salutogenic theory and relevant literature [16, 17]. We conducted an extensive literature review to search the scales and literature concerning the salutogenic theory [16, 20, 21]. Two researchers (i.e., the first and corresponding authors) summarised the relevant and possible items according to the concept model of the childbearing sense of coherence, salutogenic theory, the scales and literature concerning the salutogenic theory. These items were written in Chinese and translated into English in this article. Finally, a total of 24 items were retained in the pool of the CSOC-scale for further evaluation.

#### Delphi expert consensus

Delphi method usually requires 15–50 experts [22]. In this study, twenty-one academic experts finished two round reviews, including five males and sixteen females. They had 10 to 40 years of experience in six research areas (Clinical Nursing in Obstetrics and Gynaecology, Nursing Management, Nursing Education, Psychology, Public Health, Clinical Medicine, and other maternal health-related research areas) and from seven cities in China. A self-constructed expert advice review form was used, which helped the experts evaluate the relevance and importance of the items pool. The expert scored the importance of each item with a 5-point rating score (from 5 very important to 1 not important) and the relevance of each item with a 4-point rating score (from 4 very relevant to 1 not relevant). Experts could suggest adding, deleting, or revising items where appropriate. The criteria for deleting items were based the importance and relevance results. The Delphi stopped when the responses of experts reached stability.

#### Analysis

The mean score of the importance and Coefficient of Variation (CV) were used to evaluate the concentration and coordination of expert advice. The item is considered deleted for the mean score of importance less than 3.50 or the value of CV greater than 0.25 [22]. Content validity was calculated by the item-level content validity index (I-CVI) and scale-level content validity index (S-CVI). I-CVI was derived by dividing the number of experts rated the item as 3 or 4 by the total number of experts; S-CVI was the mean value of I-CVI of all items. A I-CVI of 0.78 or above and a S-CVI of 0.90 or above are recommended for reserving of the item [23].

#### Results of the delphi expert consensus

I-CVI ranged from 0.52 to 1.00; two items were less than 0.78; S-CVI was 0.92. Experts suggested deleting 2 items and revising the wording and expression of 17 items. The first round Delphi retained 22 items. After 14 days, the retained and revised items were sent to the same experts for review. No items were changed in the second round Delphi. All items with a CVI ranged from 0.86 to 1.00, and the S-CVI was 0.99, leaving 22 items in the preliminary scale (shown in Appendix A).

### Study 2: psychometric properties of the CSOC-scale

#### Study design and setting

This was a cross-sectional study, and data was collected using convenience sampling method. This study was conducted in four tertiary and one maternal and child health hospitals in southern, central, and northern mainland China. It was conducted during the obstetric clinic visit between December 2022 and January 2023.

#### Participants and procedures

The target participants were couples during pregnancy period. Couples’ eligibility criteria: (1) who were legal Chinese couples; (2) wives who were in singleton pregnancy; and (3) both were willing to participate in this study. Couples with: (1) one of the spouses diagnosed with psychological diseases (such as the diagnosis of depression and bipolar disorder, etc.); and (2) wives had severe obstetric comorbidities assessed from the medical records (such as severe pre-eclampsia, eclampsia, placenta previa, and chronic renal disease, etc.) [24] were excluded. Methodological scholars recommend a minimum subject-to-item ratio of at least 5:1 in exploratory factor analysis (EFA) [25]. The sample size of this study met the criteria.

The investigators were PhD and Master of Science candidates in nursing. They have all been on maternity traineeships for over a year and a half and have extensive experience communicating with pregnant women and their husbands. All investigators underwent uniform training before the formal investigation. Investigators explained the study’s aims and contents to participants. All participants provided the informed consent before the survey. Then, investigators sent each participant the self-administered online questionnaire, and the participant completed it on the mobile phone. Each individual completed the form independently. If they had any questions while completing the questionnaire, the investigator was responsible for helping to answer them.

#### Measures

Demographic information for women and men in studies 2 and 3 is presented in Table 1. The initial CSOC-scale with 22 items was used in the study.

**Table 1:** Sample characteristics

Variables	Study 2		Study 3	
	Women(*n* = 351)	Men(*n* = 256)	women(*n* = 366)	Men(*n* = 308)
Age(years)				
≤ 35	314(89.46)	226(88.28)	325(88.80)	239(77.60)
≥ 36	37(10.54)	30(11.72)	41(11.20)	69(22.40)
Ethnicity				
Han Chinese	336(95.73)	249(97.27)	357(97.54)	299(97.08)
Ethnic minorities	15(4.27)	7(2.73)	9(2.46)	9(2.92)
Education level				
Junior high school or lower	24(6.84)	11(4.30)	30(8.20)	18(5.84)
Senior high school	25(7.12)	31(12.10)	35(9.56)	36(11.69)
Bachelor’s degree/tertiary	244(69.52)	174(67.97)	248(67.76)	208(67.53)
Master’s degree or above	58(16.52)	40(15.63)	53(14.48)	46(14.94)
Occupational status				
Unemployed	85(24.22)	14(5.47)	97(26.50)	26(8.44)
Leave	48(13.68)		50(13.66)	
Employed	218(62.10)	242(94.53)	219(59.84)	282(91.56)
Monthly per capita household income (¥)				
≤ 3000	24(6.84)	8(3.13)	19(5.19)	15(4.87)
3001–5000	58(16.52)	38(14.84)	71(19.40)	50(16.23)
5001-10,000	143(40.74)	110(42.97)	159(43.44)	133(43.18)
≥ 10,001	126(35.90)	100(39.06)	117(31.97)	110(35.71)
Residence area				
Rural area	26(7.41)	20(7.81)	23(6.28)	20(6.49)
Urban area	325(92.59)	236(92.19)	343(93.72)	288(93.51)
Insurance status				
No	7(1.99)	10(3.91)	10(2.73)	12(3.90)
Yes	344(98.01)	246(96.09)	356(97.27)	296(96.10)
Weeks of gestation				
≤ 15 weeks + 6 days	22(6.27)	36(14.06)	18(4.92)	33(10.71)
16 weeks − 23 weeks + 6 days	69(19.66)	44(17.19)	84(22.95)	61(19.81)
24 weeks − 32weeks + 6 days	84(23.93)	71(27.73)	101(27.60)	75(24.35)
33 weeks - delivery	176(50.14)	105(41.02)	163(44.54)	139(45.13)
Parity				
Nullipara	261(74.36)	187(73.05)	286(78.14)	203(65.91)
Primipara	81(23.08)	51(19.92)	73(19.95)	85(27.60)
Multipara	9(2.56)	18(7.03)	7(1.91)	20(6.49)
Method of conception				
Spontaneous conception	306(87.18)	211(82.42)	310(84.70)	237(76.95)
Assisted reproductive technology	45(12.82)	45(17.58)	56(15.30)	71(23.05)

### Statistical analysis

IBM SPSS v26.0 (SPSS Inc., Chicago, IL, USA) was used for data analysis. Item analysis included item discrimination, item-total correlation, corrected item-total correlation, and scale’s Cronbach α coefficient when an item excluded [26, 27]. The total scores of the sample were ranked in descending order, with the top 27% grouped into the high-score group, and the bottom 27% into the low-score group. Two-sample *t* test was run to examine the differences in each item between the two groups, yielding critical ratio (CR) values, which should be over than 3.00 and *P* < 0.05 [27]. Item-total correlation over than 0.40 met the criteria [27]. Corrected item-total correlation less than 0.400 should be deleted [27]. If the scale’s Cronbach α coefficient increased upon the exclusion of an item, the item should be deleted [27, 28]. Construct validity was evaluated by exploratory factor analysis (EFA). The principal axis method using orthogonal rotation (equal-maximum method) was performed on study 2 sample (*N* = 351 for women, *N* = 256 for men). Items with factor loadings less than 0.400 or cross-loadings greater than 0.450 and a difference less than 0.200 were excluded [29]. Before conducting EFA, we need to judge whether the sample data are suitable for EFA from the Kaiser-Meyer-Olkin measure of sampling adequacy (KMO) and Barllett’s test of sphericity. The value of KMO is between 0 and 1, the closer to 1 indicates that the relationship between the items is better, the more suitable for factor analysis; the value greater than 0.90 indicates it is very suitable. Barllett’s test of sphericity was used to test the independence of the variables; if *P* < 0.05 indicates that the values of the variables are correlated, it is suitable for factor analysis.

## Results

The 22-item scale was piloted on 50 couples (50 females and 50 males) during their antenatal obstetric visit or waiting for delivery in the obstetric ward in one of the hospitals where the formal study was conducted. The initial assessment of participants on items’ descriptions was good, and the items were not changed. Then, we collected data from the target population for item analysis and EFA. Table 1 presented the characteristics of participants (*N* = 351 for women, *N* = 256 for men).

### Item analysis

For item discrimination, CR for all items was over 3.00 and *P* < 0.05 for women and men. For item-total correlation, item B1 was less than 0.40 in women, and all items were more than 0.40 in men. For women, the scale’s Cronbach α coefficient was 0.943; the corrected item-total correlation of items B1 and B2 were both less than 0.400. When B1 and B2 was excluded, the scale’s Cronbach α coefficient were 0.947 and 0.946, respectively, both above 0.943. For men, the scale’s Cronbach α coefficient was 0.938; the corrected item-total correlation of item B1 was less than 0.400. When B1 or B2 was excluded, the scale’s Cronbach α coefficient were 0.942 and 0.940, respectively, both above 0.938. Based on the criteria of item analysis, items B1 and B2 were eliminated for women and men. The detailed item analysis results were shown in Table 2 for women and Table 3 for men. The scale retained 20 items in both males and females.

**Table 2:** Item analysis for women (*N* = 351)

Item number	Item discrimination	Item-total correlation	Corrected item-total correlation	Scale’s Cronbach α coefficient when an item excluded	Item exclusion or retention
A1	17.679***	0.768^**^	0.738	0.939	retained
A2	15.875***	0.784^**^	0.761	0.939	retained
A3	15.797***	0.803^**^	0.779	0.939	retained
A4	17.455***	0.809^**^	0.787	0.939	retained
A5	16.753***	0.782^**^	0.755	0.939	retained
A6	15.920***	0.741^**^	0.708	0.940	retained
A7	13.890***	0.737^**^	0.706	0.940	retained
A8	13.529***	0.709^**^	0.675	0.940	retained
B1	6.052***	0.346^**^	0.271	0.947	excluded
B2	6.934***	0.400^**^	0.327	0.946	excluded
B3	10.235***	0.568^**^	0.514	0.943	retained
B4	9.585***	0.501^**^	0.445	0.943	retained
B5	13.550***	0.625^**^	0.577	0.942	retained
B6	10.665***	0.516^**^	0.457	0.943	retained
C1	14.673***	0.713^**^	0.684	0.940	retained
C2	16.841***	0.794^**^	0.769	0.939	retained
C3	16.007***	0.723^**^	0.686	0.940	retained
C4	17.639***	0.792^**^	0.768	0.939	retained
C5	17.554***	0.795^**^	0.772	0.939	retained
C6	16.446***	0.744^**^	0.711	0.940	retained
C7	17.578***	0.784^**^	0.755	0.939	retained
C8	16.523***	0.819^**^	0.799	0.939	retained

*P < 0.05, ** *P* < 0.01, *** *P* < 0.001

**Table 3:** Item analysis for men (*N* = 256)

Item number	Item discrimination	Item-total correlation	Corrected item-total correlation	Scale’s Cronbach α coefficient when item excluded	Item exclusion or retention
A1	16.088***	0.797***	0.774	0.933	retained
A2	12.871***	0.773***	0.751	0.934	retained
A3	13.138***	0.811***	0.790	0.933	retained
A4	14.845***	0.780***	0.754	0.933	retained
A5	15.809***	0.791***	0.767	0.933	retained
A6	14.714***	0.690***	0.654	0.934	retained
A7	13.353***	0.695***	0.658	0.934	retained
A8	12.144***	0.700***	0.663	0.934	retained
B1	11.874***	0.453***	0.369	0.942	excluded
B2	13.887***	0.525***	0.445	0.940	excluded
B3	10.313***	0.556***	0.501	0.937	retained
B4	9.739***	0.571***	0.515	0.937	retained
B5	9.388***	0.608***	0.552	0.936	retained
B6	10.992***	0.601***	0.543	0.937	retained
C1	12.725***	0.746***	0.718	0.934	retained
C2	14.242***	0.757***	0.730	0.934	retained
C3	14.807***	0.722***	0.693	0.934	retained
C4	15.205***	0.743***	0.716	0.934	retained
C5	12.763***	0.733***	0.705	0.934	retained
C6	11.071***	0.638***	0.594	0.935	retained
C7	15.035***	0.745***	0.713	0.933	retained
C8	10.887***	0.741***	0.713	0.934	retained

*P < 0.05, ** *P* < 0.01, *** *P* < 0.001

### Exploratory factor analysis

**Fig. 1:**
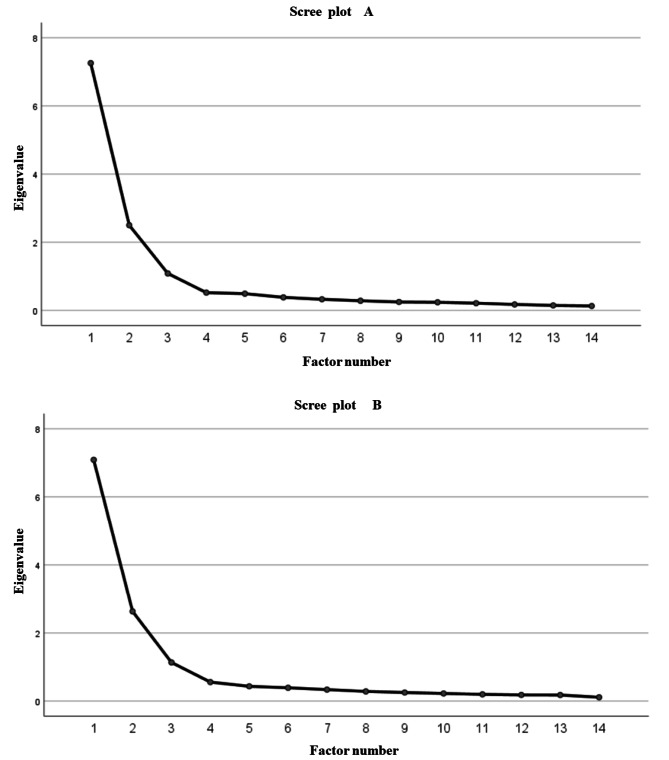
Scree plot and eigenvalue of exploratory factor analysis for women **(A)** and men **(B)**

In the first time EFA, the KMO test was 0.946 for women and 0.937 for men, and Bartlett’s test of sphericity was 6660.540 for women and 4889.918 for men, with *P* < 0.001, which was suitable for factor analysis. In further EFA, items A1, A2, A3, A4, C7, and C8 were deleted for both women and men due to violation of the criteria for item retention of EFA. After the seventh exploratory factor analysis for both women and men, a total of 14 items met the criteria for factor analysis, and three factors with eigenvalues greater than 1 were extracted (Fig. 1A for women, Fig. 1B for men), explaining 71.53% and 71.77% of the variance of the full scale for women and men, respectively (Table 4). Factor loading ranged from 0.646 to 0.851 for women and 0.611 to 0.862 for men. The factors were labelled according to the concept model of the childbearing sense of coherence, including three factors. The first factor included items q1 to q4 (referring to the extent to which the individual regards the stimuli from the internal and external environments during childbearing as reasonable and acceptable), being labelled “comprehensibility of childbearing”. Factor two included items q5 to q8 and was labelled “manageability of childbearing”, as the items represent the extent to which the individual utilises internal and external resources to cope with challenges from childbearing. Factor three (items q9 to q14) addressed the extent to which the individual recognises the meaningfulness of everything experienced during childbearing and is willing to put effort into it, and was therefore labelled the “meaningfulness of childbearing”.

**Table 4:** Exploratory factor analysis of the childbearing sense of coherence (*N* = 351 for women, *N* = 256 for men)

	Factor loadings					
items	Women			Men		
	Factor 1	Factor 2	Factor 3	Factor 1	Factor 2	Factor 3
A5(q1). I am able to accept the impact of childbearing on my personal interests	**0.735**	0.103	0.380	**0.682**	0.170	0.379
A6(q2). I am able to accept the impact of childbearing on my career development	**0.829**	0.138	0.325	**0.809**	0.084	0.323
A7(q3). I am able to accept the impact of childbearing on my personal interactions with people	**0.840**	0.117	0.333	**0.862**	0.103	0.305
A8(q4). I am able to accept the impact of childbearing on my personal life	**0.813**	0.101	0.316	**0.798**	0.118	0.319
B3(q5). I often felt frustrated during the childbearing process (R)	0.037	**0.809**	0.170	0.011	**0.814**	0.149
B4(q6). I often felt treated unfairly during the childbearing process (R)	0.050	**0.766**	0.118	0.141	**0.834**	0.064
B5(q7). I often felt overwhelmed with responsibilities during the childbearing process (R)	0.141	**0.851**	0.193	0.076	**0.857**	0.139
B6(q8). I often doubted my own abilities during the childbearing process (R)	0.146	**0.815**	0.041	0.134	**0.823**	0.105
C1(q9). Childbearing makes me grow	0.343	0.169	**0.646**	0.297	0.190	**0.760**
C2(q10). Childbearing gives me happiness	0.334	0.193	**0.765**	0.351	0.188	**0.778**
C3(q11). Childbearing makes me motivated	0.306	0.160	**0.711**	0.301	0.148	**0.766**
C4(q12). Childbearing makes my life fulfillment	0.393	0.128	**0.774**	0.362	0.148	**0.772**
C5(q13). Childbearing strengthens my family bond	0.345	0.241	**0.731**	0.374	0.139	**0.718**
C6(q14). Childbearing makes my life extended	0.374	0.127	**0.709**	0.350	0.057	**0.611**
Eigenvalues	3.375	2.865	3.774	3.239	2.969	3.841
Accounting of variance (%)	24.109	20.468	26.957	23.135	21.205	27.433
Cumulated variance (%)	24.109	44.577	71.533	23.135	44.340	71.773

(R) reverse-coded item;

### Study 3: reliability and validity of the CSOC-scale

Study design and setting, inclusion and exclusion criteria for participants, study procedures, and measures were the same as in Study 2. This study was conducted between January 2023 and February 2023.

#### Instruments

The 14-item CSOC-scale which had been previously subjected to item analysis and EFA in study 2, was used to evaluate the women’s and men’s childbearing sense of coherence. It consists of three dimensions (comprehensibility of childbearing containing four items; the manageability of childbearing containing four items; the meaningfulness of childbearing containing six items). Each item uses a five-point Likert score, ranging from 1 (strongly disagree) to 5 (strongly agree). Items in manageability of childbearing are reverse scored.

### Statistical analysis

Maximum-likelihood method (ML) was performed in CFA using Mplus Version 8.3. All items conformed to normality (skewness and kurtosis below the thresholds of 2 and 7, respectively) [25]. The values of indices to estimate the goodness-of-fit were *χ*^*2*^/ *df*, Root Mean Square Error of Approximation (RMSEA), Comparative Fit Index (CFI), Tucker-Lewis index (TLI), and Standardised Root Mean Square Residual (SRMR). An acceptable fit is indicated by 2 < *χ*^*2*^/ *df* ≤ 3, 0.050 < RMSEA ≤ 0.080, 0.950 ≤ CFI < 0.970, 0.950 ≤ TLI < 0.970, 0.050 < SRMR ≤ 0.100 [30, 31]. Cronbach’s α and split-half reliability were used to measure the reliability. The value of reliability coefficients greater than 0.800 was considered good and greater than 0.700 was considered acceptable [27, 28].

## Results

### Confirmatory factor analysis

**Fig. 2:**
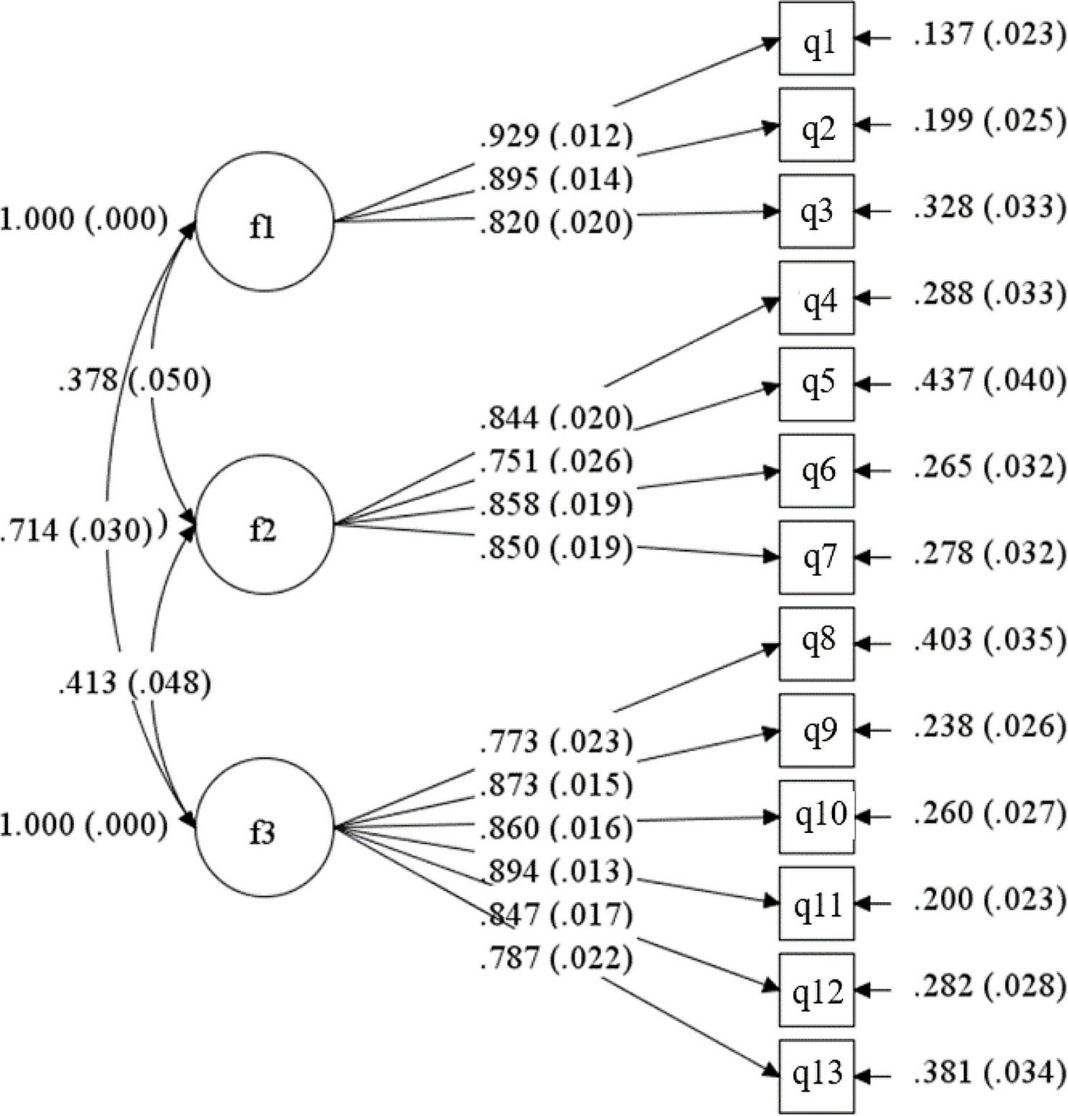
Confirmatory factor analysis factor loadings for the 13-item childbearing sense of coherence scale in women (*N* = 366). Note: all factor loadings are standardized all *P* < 0.001; fitness indices: *χ*^*2*^ *= 157.448, df = 62, χ*^*2*^*/ df = 2.539, RMSEA = 0.065, CFI = 0.974, TLI = 0.968, SRMR = 0.029*

Table 1 presented the characteristics of participants (Study 3, *N* = 366 for women, *N* = 308 for men). Model fit indices of CFA in women were *χ*^*2*^ = 280.197, *df* = 74, *χ*^*2*^/ *df* = 3.786, RMSEA = 0.087, CFI = 0.952, TLI = 0.940, SRMR = 0.031. The values of *χ*^*2*^/ *df* and RMSEA were not very ideal. The model was adjusted according to modification indices (MI), which showed that item q3 had the most significant correlation coefficient with q4, with a value of 85.357. We deleted item q3, considering that the coverage of item q4 was more comprehensive than item q3. Another round of CFA demonstrated good fit as indicated by multiple model fit indices (*χ*^*2*^ = 157.448, *df* = 62, *χ*^*2*^/ *df* = 2.539, RMSEA = 0.065, CFI = 0.974, TLI = 0.968, SRMR = 0.029). Factor one loadings ranged from 0.820 to 0.929; factor two loadings ranged from 0.751 to 0.858; factor three loadings ranged from 0.773 to 0.894, all factor loadings with *P* < 0.001. After deletion of the item, the items of the final scale were renumbered in the order in which they appeared. Figure 2 displayed the standardised factor loadings of CFA for women.

**Fig. 3:**
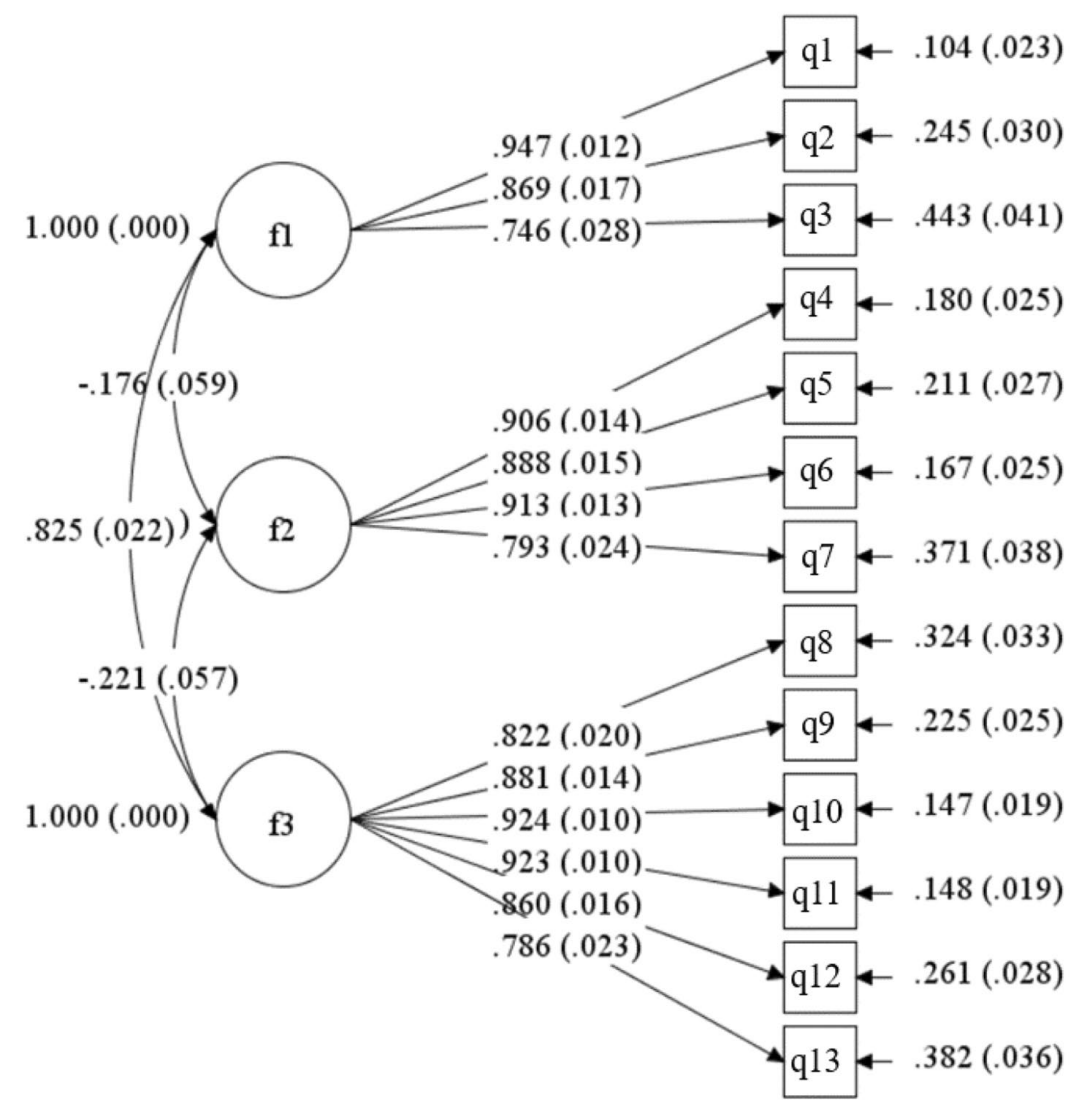
Confirmatory factor analysis factor loadings for the 13-item childbearing sense of coherence scale in men (*N* = 308). Note: all factor loadings are standardized all *P* < 0.001; fitness indices: *χ*^*2*^ = 181.363, *df* = 62, *χ*^*2*^/ *df* = 2.925, RMSEA = 0.079, CFI = 0.968, TLI = 0.960, SRMR = 0.033

Model fit indices of CFA in men were *χ*^*2*^ = 336.531, *df* = 74, *χ*^*2*^/ *df* = 4.548, RMSEA = 0.107, CFI = 0.937, TLI = 0.922, SRMR = 0.037. The values of *χ*^*2*^/ *df* and RMSEA were not acceptable. MI showed that item q3 had the largest correlation coefficient with q4, with a value of 128.323. We deleted item q3 with the same reason in women. Another round of CFA demonstrated good model fit indices (*χ*^*2*^ = 181.363, *df* = 62, *χ*^*2*^/ *df* = 2.925, RMSEA = 0.079, CFI = 0.968, TLI = 0.960, SRMR = 0.033). Factor one loadings ranged from 0.746 to 0.947; factor two loadings ranged from 0.793 to 0.913; factor three loadings ranged from 0.786 to 0.924, all factor loadings with *P* < 0.001. After deletion of the item, the items of the final scale were renumbered in the order in which they appeared. Figure 3 demonstrated the standardised factor loadings of CFA for men

### Reliability and validity

Table 5 demonstrated the reliability of the final CSOC-scale. For the CSOC-scale, Cronbach’s α was 0.919 for women and 0.821 for men, and split-half reliability was 0.744 for women and 0.785 for men. For dimension 1 (comprehensibility of childbearing), Cronbach’s α was 0.912 for women and 0.881 for men, and split-half reliability was 0.885 for women and 0.841 for men. For dimension 2 (manageability of childbearing), Cronbach’s α was 0.895 for women and 0.928 for men, and split-half reliability was 0.876 for women and 0.909 for men. For dimension 3 (meaningfulness of childbearing), Cronbach’s α was 0.933 for women and 0.945 for men, and split-half reliability was 0.921 for women and 0.937 for men. The final CSOC-scale were presented in Appendix B.

**Table 5:** Reliability coefficients (Cronbach’s α and split-half reliability) for the CSOC-scale (*N* = 366 for women, *N* = 308 for men)

Scale/Dimensions	No. of items	Cronbach’s α		Split-half reliability	
		Women	Men	Women	Men
Childbearing sense of coherence	13	0.919	0.821	0.744	0.785
Comprehensibility of childbearing	3	0.912	0.881	0.885	0.841
Manageability of childbearing	4	0.895	0.928	0.876	0.909
Meaningfulness of childbearing	6	0.933	0.945	0.921	0.937

## Discussions

In current study, we proposed three factors of the CSOC-scale (comprehensibility of childbearing, manageability of childbearing, and meaningfulness of childbearing), which is consistent with the original concept of it. The scale comprises 13 items with a five-point Likert score (ranging from strongly disagree = 1 to strongly agree = 5). It measures individuals’ current childbearing sense of coherence. The total score ranged from 13 to 65, with higher scores indicating higher levels of childbearing sense of coherence. The CSOC-scale demonstrated good content validity, construct validity, and sound reliability.

In the context of childbearing, compared with the FSOC scale and SOC scale, the CSOC-scale included items about childbearing, which would be more targeted to be used in the specific context of childbearing. For the first dimension (comprehensibility of childbearing), the items concerning the extent to which one could accept the impact of childbearing on one’s interests, personal life, and career development. These findings were consistent with some previous studies [32]. In a qualitative study conducted among 16 pregnant and 10 postpartum women in the USA, Mahaffey et al. found that many employed women worried that pregnancy-related fatigue, maternity leave, and parenting time demands would influence productivity at work; some participants expressed concerns that they would lose other identities due to the new identity of the mother [33]. In another qualitative study among first-time fathers from six different ethnic backgrounds in the UK, participants expressed difficulties in balancing work, home life, and themselves [6]. Concurrent stresses, such as those related to work, personal life, or family roles, may exacerbate the stress of pregnancy on couples during the parenthood transition [32]. However, current research does not have a measurement tool to quantify this parenthood shift in triggering pressure. Therefore, the measure of comprehensibility of childbearing could help healthcare professionals evaluate the extent of one’s acceptance of the impact of childbearing so that they can target their health promotion strategies.

The second dimension (manageability of childbearing) refers to the extent to which the individual utilises internal and external resources to cope with challenges from childbearing, items containing feelings of frustration, being treated unfairly, overwhelming responsibilities, and doubting own abilities during the childbearing process. Figuring out internal and external resources can help parents in transition improve their ability to cope with challenges and pressures. Shortage of high-quality and reliable health information and lower levels of health literacy and parenting skills were the main causes of loss of control and autonomy in one’s body or life and feelings of uselessness among both women and men during the parenthood transition [6, 33]. These findings suggest that internal resources, such as health literacy and experience, and external resources, like support from family, friends, experienced parents, and healthcare professionals, are critical for couples to cope with the challenges of childbearing [8, 10, 34, 35]. Unlike many previous studies that focused mainly on women, our study sample included both women and men, highlighting that the health of both genders should be considered in the perinatal period. A good cycle is when fathers have good health status in the perinatal period; in turn, they can provide appropriate emotional and behavioural support to their wives [36]. More importantly, understanding the level of an individual’s manageability of childbearing can inform the provision of effective support from internal and external resources for parents in transition.

The third dimension (meaningfulness of childbearing) refers to the extent to which the individual recognises the meaningfulness of everything experienced during childbearing and is willing to put effort into it. It included items on personal realisation, family bonding and harmony, and the continuation of life. These findings were similar to previous studies. In a qualitative study in the UK from six ethnic backgrounds, fathers expressed that new fatherhood brought them a sense of accomplishment and growth [6]. In addition, fathers said the positive aspect of new parenthood is that their relationship with their wives was stronger than before; some described that these changes negatively affected their relationship with their partner due to having less time to spend with their partners and being more irritable of their partners [6]. The meaningfulness of family bonding and harmony may be especially crucial and have special meaning for Chinese influenced by Confucianism [17]. Xiao (filial piety) is an essential tenet of Confucianism. A classic old saying that has been widely circulated is that there are three forms of unfilial conduct, of which the worst is to have no descendants [37]. The meaningfulness of family bonding and harmony includes the relationship between husband and wife as well as the relationship with parents among Chinese. The further study can compare whether there are differences in the dimensions of meaningfulness of childbearing across cultures.

The construct validity was evaluated using factor analysis. Both EFA and CFA in women and men samples achieved values that satisfied published recommendations [25]. Cronbach α and split-half reliability were calculated to assess internal consistency, and both of the coefficients were high in women and men. Given that it is brief and has 13 items, it will be used efficiently and cause little burden to participants. This implies that this tool could measure the perspective of interrelated couples. Couples as protagonists of childbearing, the perception of the CSOC of one partner may be associated with comprehensibility of childbearing, manageability of childbearing, and meaningfulness of childbearing of another partner. In addition, the salutogenic theory is reported to play an important role in maternal and paternal health during parental transition [12, 38]. Therefore, the CSOC-scale could provide the measures for clinical practice in real-world settings to evaluate the childbearing sense of coherence among couples during the perinatal period. The evaluation could inform healthcare providers, managers, and policymakers on the direction to make health-promoting strategies by adhering to the subcomponents of the childbearing sense of coherence framework to assist couples during the transition to parenthood.

### Limitations and strengths

First, this study used a convenience sampling method, which may limit the generalizability of the results in the present study. Replicating the findings in other samples, even in different cultures, is desirable. Second, in this study, the sample in studies 2 and 3 was pregnant couples. Further study is recommended to validate the CSOC-scale in couples in other perinatal periods, such as postpartum and pre-pregnancy. Third, the participants included in this study was not strictly controlled for pregnant period, parity, and method of conception, which could affect the childbearing sense of coherence. Future studies could be applied with the participants in a single group or the groups could be included in approximate numbers. The strength of this study is that the sample was from 5 hospitals in southern, central, and northern mainland China, and the scale can be applied to men and women. In addition, the sample size in this study is more than 10 participants per item for the requirement of factor analysis.

## Conclusions

In this study, we developed the CSOC-scale based on the salutogenic theory and the concept of childbearing sense of coherence from the individual level and childbearing orientation. It is assumed to evaluate the individuals’ ability to maintain health in childbearing aspects. The results suggest that the CSOC-scale has three dimensions and thirteen items with robust psychometric properties in both genders during pregnancy. Utilisation of this scale can help healthcare professionals understand couples’ health maintenance competencies during the transition of parenthood and provide health promotion services from a salutogenic perspective.

### Electronic supplementary material

Below is the link to the electronic supplementary material.

Supplementary Material 1

Supplementary Material 2

## Data Availability

The data used in the current study are available from the corresponding author upon reasonable request.

## Abbreviations

SOC: Sense of Coherence
FSOC: the Family Sense of Coherence Scale
CSOC: Childbearing Sense of Coherence
CV: Coefficient of Variation
I-CVI: the Item-level Content Validity Index
S-CVI: Scale-level Content Validity Index
ML: Maximum-likelihood Method
RMSEA: Root Mean Square Error of Approximation
CFI: Comparative Fit Index
TLI: Tucker-Lewis index
SRMR: Standardised Root Mean Square Residual
CR: Critical Ratio
EFA: Exploratory Factor Analysis
CFA: Confirmatory Factor Analysis
KMO: the Kaiser-Meyer-Olkin Measure of Sampling Adequacy
MI: Modification Indices
