# Supplementary material for: The Childbearing sense of coherence scale (CSOC-scale): development and validation
Source: BMC Public Health. 2024 Jun 17;24:1613. doi: 10.1186/s12889-024-19109-1 (PMC11181555; doi:10.1186/s12889-024-19109-1)
Supplement: Supplementary file 1 — Supplementary Material 1 [file 12889_2024_19109_MOESM1_ESM.docx]

| Factors | Items tool |
| --- | --- |
| Comprehensibility of childbearing | A1. I am able to afford to take the health risks of childbearing |
|  | A2. I am able to take the responsibility of childbearing |
|  | A3. I am able to withstand the hardship of childbearing |
|  | A4. I am able to accept the impact of childbearing on family relationships |
|  | A5. I am able to accept the impact of childbearing on my personal interests |
|  | A6. I am able to accept the impact of childbearing on my career development |
|  | A7. I am able to accept the impact of childbearing on my personal interactions with people |
|  | A8. I am able to accept the impact of childbearing on my personal life |
| Manageability of childbearing | B1. I often felt out of control during the childbearing process. |
|  | B2. I often felt helpless during the childbearing process. |
|  | B3. I often felt frustrated during the childbearing process. |
|  | B4. I often felt treated unfairly during the childbearing process. |
|  | B5. I often felt overwhelmed with responsibilities during the childbearing process. |
|  | B6. I often doubted my own abilities during the childbearing process. |
| Meaningfulness of childbearing | C1. Childbearing makes me grow. |
|  | C2. Childbearing gives me happiness. |
|  | C3. Childbearing makes me motivated. |
|  | C4. Childbearing makes my life fulfillment |
|  | C5. Childbearing strengthens my family bond. |
|  | C6. Childbearing makes my life extended. |
|  | C7. I think it's worth it for what I'm going through during the childbearing process. |
|  | C8. I'm willing to put in the effort for childbearing. |
