# Supplementary material for: The Childbearing sense of coherence scale (CSOC-scale): development and validation
Source: BMC Public Health. 2024 Jun 17;24:1613. doi: 10.1186/s12889-024-19109-1 (PMC11181555; doi:10.1186/s12889-024-19109-1)
Supplement: Supplementary file 2 — Supplementary Material 2 [file 12889_2024_19109_MOESM2_ESM.docx]

| Factors | Items | Scaling | | | | |
| --- | --- | --- | --- | --- | --- | --- |
|  |  | Strongly disagree | disagree | Neither disagree or agree | agree | Strongly agree |
| Comprehensibility of childbearing | 1. I am able to accept the impact of childbearing on my personal interests |  |  |  |  |  |
|  | 2. I am able to accept the impact of childbearing on my career development |  |  |  |  |  |
|  | 3. I am able to accept the impact of childbearing on my personal life |  |  |  |  |  |
| Manageability of childbearing | 4. I often felt frustrated during the childbearing process. (R) |  |  |  |  |  |
|  | 5. I often felt treated unfairly during the childbearing process. (R) |  |  |  |  |  |
|  | 6. I often felt overwhelmed with responsibilities during the childbearing process. (R) |  |  |  |  |  |
|  | 7. I often doubted my own abilities during the childbearing process. (R) |  |  |  |  |  |
| Meaningfulness of childbearing | 8. Childbearing makes me grow. |  |  |  |  |  |
|  | 9. Childbearing gives me happiness. |  |  |  |  |  |
|  | 10. Childbearing makes me motivated. |  |  |  |  |  |
|  | 11. Childbearing makes my life fulfillment |  |  |  |  |  |
|  | 12. Childbearing strengthens my family bond. |  |  |  |  |  |
|  | 13. Childbearing makes my life extended. |  |  |  |  |  |

Note scoring: items with (R) are reverse-coded item; strongly disagree=1, disagree=2, neither disagree or agree=3, agree=4, strongly agree=5. Scores for each of three dimensions are calculated as follows. Comprehensibility of Childbearing: sum of items 1, 2, and 3; Manageability of Childbearing: sum of items 4, 5, 6, and 7. Meaningfulness of Childbearing: sum of items 8, 9, 10, 11, 12, and 13. The total score ranged from 13 to 65, with higher score indicating higher levels of childbearing sense of coherence.
